# CRISPRCasdb a successor of CRISPRdb containing CRISPR arrays and *cas* genes from complete genome sequences, and tools to download and query lists of repeats and spacers

**DOI:** 10.1093/nar/gkz915

**Published:** 2019-10-18

**Authors:** Christine Pourcel, Marie Touchon, Nicolas Villeriot, Jean-Philippe Vernadet, David Couvin, Claire Toffano-Nioche, Gilles Vergnaud

**Affiliations:** 1 Institute for Integrative Biology of the Cell (I2BC), CEA, CNRS, Univ. Paris-Sud, Université Paris-Saclay, 91198 Gif-sur-Yvette, France; 2 Microbial Evolutionary Genomics, Institut Pasteur, 25–28 rue du Docteur Roux, 75015 Paris, France; 3 CNRS, UMR3525, 25–28 rue du Docteur Roux, 75015 Paris, France; 4 Unité Transmission, Réservoir et Diversité des Pathogènes, Institut Pasteur de Guadeloupe, 97139 Les Abymes, France

## Abstract

In Archaea and Bacteria, the arrays called CRISPRs for ‘clustered regularly interspaced short palindromic repeats’ and the CRISPR associated genes or *cas* provide adaptive immunity against viruses, plasmids and transposable elements. Short sequences called spacers, corresponding to fragments of invading DNA, are stored in-between repeated sequences. The CRISPR–Cas systems target sequences homologous to spacers leading to their degradation. To facilitate investigations of CRISPRs, we developed 12 years ago a website holding the CRISPRdb. We now propose CRISPRCasdb, a completely new version giving access to both CRISPRs and *cas* genes. We used CRISPRCasFinder, a program that identifies CRISPR arrays and *cas* genes and determine the system's type and subtype, to process public whole genome assemblies. Strains are displayed either in an alphabetic list or in taxonomic order. The database is part of the CRISPR-Cas^++^ website which also offers the possibility to analyse submitted sequences and to download programs. A BLAST search against lists of repeats and spacers extracted from the database is proposed. To date, 16 990 complete prokaryote genomes (16 650 bacteria from 2973 species and 340 archaea from 300 species) are included. CRISPR–Cas systems were found in 36% of Bacteria and 75% of Archaea strains. CRISPRCasdb is freely accessible at https://crisprcas.i2bc.paris-saclay.fr/.

## INTRODUCTION

Clustered regularly interspaced short palindromic repeats (CRISPRs) have been described in a wide range of prokaryotes, including 80% of Archaea and 40% of Bacteria strains for which complete genome sequences were available (1). They consist in the succession of 23–50 bp repeated sequences (often called direct repeats or DR) separated by unique sequences of a similar length called spacers (2–5). The spacers correspond to fragments of foreign DNA originating mostly from viruses and other mobile genetic elements (6–10). Usually, a cluster of genes called *cas* for CRISPR-associated is located in the vicinity of a CRISPR, and together they form a CRISPR–Cas system (11). When several CRISPRs with the same repeat are present at different positions along the chromosome, only one cluster of *cas* genes is found, flanked by one or two CRISPR arrays. Cas proteins play a role in the three steps of the CRISPR–Cas immunity, the acquisition of new spacers, the maturation of a long CRISPR transcript into small crRNAs and the interference which is the targeting and cleavage of the invading genome (mostly DNA but also RNA in some systems). CRISPR–Cas systems are of great interest both for basic research and biotechnological developments. There is a large diversity of Cas clusters (12) and thorough analysis of available genomes allowed to distribute them into two classes, six types (I to VI) and 33 subtypes (13,14). Class 1 systems necessitate a group of Cas to perform interference, whereas in the class 2 systems interference is performed by a large multifunction protein such as Cas9, Cas12 and Cas13 in type II, type V and type VI systems respectively (13,15). CRISPR–Cas systems can be found on plasmids and are often associated with transposable elements allowing their transfer between strains and species (16).

Dedicated software have been developed to detect CRISPR arrays, the most used being PILER-CR (17), CRT (18), CRISPRFinder (19) and MinCED (https://github.com/ctSkennerton/minced). Detection of Cas proteins is performed either by BLAST or by Hidden Markov Model (HMM) search such as in CasFinder, a development of MacSyFinder (20) and HMMCAS (21), using a set of reference Cas from well-known systems. Defining accurately the CRISPR–Cas systems remains a challenge when new genomes are analysed, particularly for the detection of Cas9-like proteins which show a high degree of diversity (22–24). HMM protein profiles such as those available at TIGRFAM (25) or Pfam sites (26) need to be improved when new sequences become available.

Databases that can be queried online are valuable tools to investigate the diversity of the CRISPR–Cas systems, if they are regularly updated. Following the development of the CRISPRFinder program, we launched the first website dedicated to these structures and holding a database called CRISPRdb (27). Recently we created a new website and developed a tool called CRISPRCasFinder which associates CRISPRFinder and CasFinder to identify both CRISPR arrays and *cas* genes in submitted sequences (28). Other databases were created such as CRISPI (29), CRISPRBank (30), CRISPRone (31) and CRISPRminer (32). CRISPRDetect is a program that can be used locally or online to analyse genome sequences (30), whereas CRISPRdisco allows the discovery and analysis of CRISPR–Cas systems by installing the program locally (33). We now describe CRISPRCasdb, the database built with CRISPRCasFinder and we compare it to other available online databases.

## MATERIALS AND METHODS

### Database and software design and implementation

CRISPRCasdb and associated services are implemented in Microsoft .Net Core 2.2 (multiplatform web application framework), PostgreSQL 9.5 (RDBMS) and Python (database feeding and updates, BLAST jobs management). Both database and web server run on a single 4-cores virtual machine, while a physical server with 64 cores and 128Gb of memory provides the calculation part for CRISPR and Cas detection and BLAST jobs (34). Both machines run in a Linux environment (Ubuntu 16.04).

The core application consists of two main programs: CRISPRCasFinder to detect CRISPRs and *cas* genes and extract them from a genomic sequence, and ‘Database Tools’ for downloading prokaryotic genomes, metadata and taxonomy from the NCBI ftp site, running CRISPRs and Cas detection scripts on downloaded sequences, storing results, and allowing BLAST searches on DRs and spacers stored in the database. CRISPRCasFinder is a full command line tool written in-house in Perl. It is used to process published genome sequences and to feed the CRISPRCas database. It can also be run interactively through the web interface for submission and analysis of users sequence data (28). ‘Database Tools’ are a set of Python and Perl scripts (the workflow is shown in Supplementary Figure S1). Downloading of genomic sequences, CRISPRs and Cas detection, and motifs extraction are fully automated.

The .Net Core Framework, providing a set of tools for object-oriented web programming and an integrated web server is used to build a web resource on top of these programs. This preserves platform independence across multiple operating systems and allows the user to interact with the different CRISPR tools programs without computer programming or (shell) scripting skills.

### The database (CRISPRCasdb)

**Figure 1.**
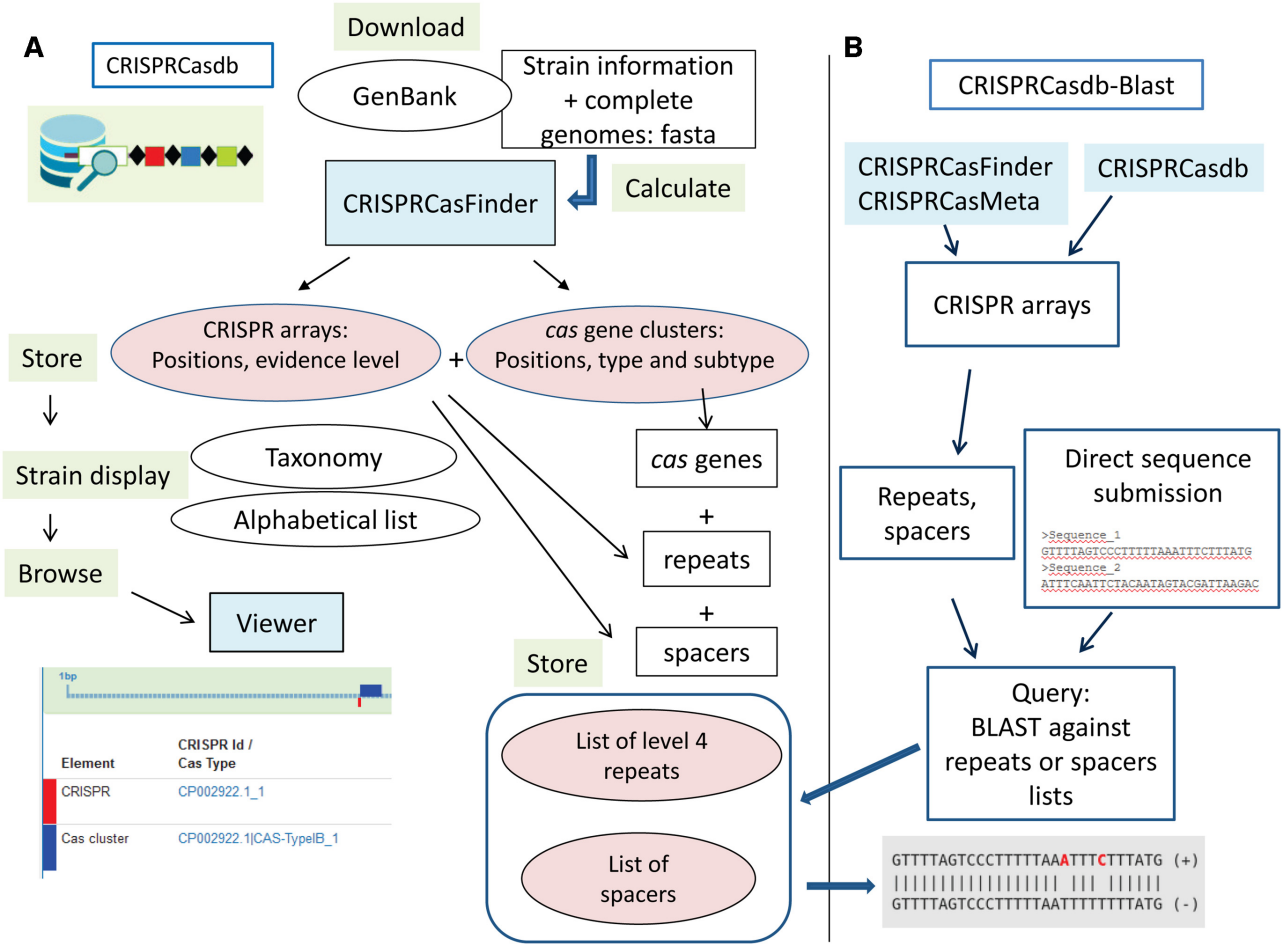
Workflow for the development of CRISPRCasdb. (**A**) Workflow for the recovery of genome sequences and associated data, CRISPRCasFinder calculation, storage and display of data. (**B**) Implementation of CRISPRCasdb-BLAST. Sequences provided in the output of CRISPRCasdb, CRISPRCasFinder, CRISPRCasMeta or directly submitted by users can be blasted against lists of repeats and spacers from the database.

CRISPRCasdb is a relational database implemented using postsgreSQL 9.5. The flowchart on Figure 1A summarizes the different steps of the database constitution. Supplementary Figure S2 shows the Unified Modeling Language (UML) class diagram, and Supplementary Figure S3 shows the tables interactions. Currently, CRISPRCasdb is composed of 15 tables. A BLAST search against lists of repeats and spacers has been implemented (Figure 1B).

The database is regularly updated by adding newly available genomes, and a version of the updater scripts allowing weekly update is being developped. If a major evolution of the CRISPRCasFinder program or associated HMM profiles is released, all the available genomes are downloaded and re-analysed when updating the database. This allows regularly improving the definition of structures when new Cas types and subtypes are defined.

In June 2019, all ‘complete genome’ and ‘chromosome’ publicly available in GenBank were recovered from NCBI (35) together with taxonomy information (36), and the database was built using CRISPRCasFinder v4.2.19 program. The selected criteria require that the minimal structure of a putative CRISPR should consist in at least two successive direct repeats with a maximum of one mismatch, separated by one spacer. Tests are performed to classify the putative CRISPRs arrays with evidence level 1 to 4. CRISPRs of less than 4 spacers with three or more perfect repeats are assigned the lowest evidence level. The other CRISPRs are classified based on the conservation of repeats which must be high in a real CRISPR array, and on the similarity between spacers which must be low. We measure CRISPR repeat conservation based on Shannon's entropy and produce an EBcons (entropy-based conservation) index (28). Level 4 CRISPRs are the most reliable ones and levels 1, 2 and 3 must be considered with caution as they may correspond to false CRISPRs. Putative Cas proteins are searched by sequence similarity using HMM protein profiles (15,23). The assignment of a protein to a given subtype is decided based on its compliance with the content and organization defined in each model (one by subtype) of CasFinder v2.0.3 (20,28). Subtypes of class 1 systems are detected from three genes, while class 2 systems necessitate a single signature gene. Thus, if a class 1 *cas* gene cluster contains less than three genes, or if a cluster has an atypical content or organization, no subtype can be determined. In addition, if the content of the cluster is not informative enough to accurately determine the subtype, the system is called CAS. CRISPR arrays and *cas* clusters are detected independently of each other. Therefore, CRISPR are indicated, whether or not *cas* genes are present, and vice-versa.

A dump of the database content, and lists of consensus repeats and spacers are provided on the website for download.

## RESULTS

The CRISPR–Cas++ website which holds the CRISPRCasdb and associated tools was designed to allow fast analyses and updates of the site. In the current version of the database, only complete genomes of bacteria and archaea strains were analysed. Downloading of the genomes and metadata from 16 990 strains (available on June 2019), and CRISPRCasFinder calculation were achieved in 150 h.

### CRISPRCasdb: display and query

**Figure 2.**
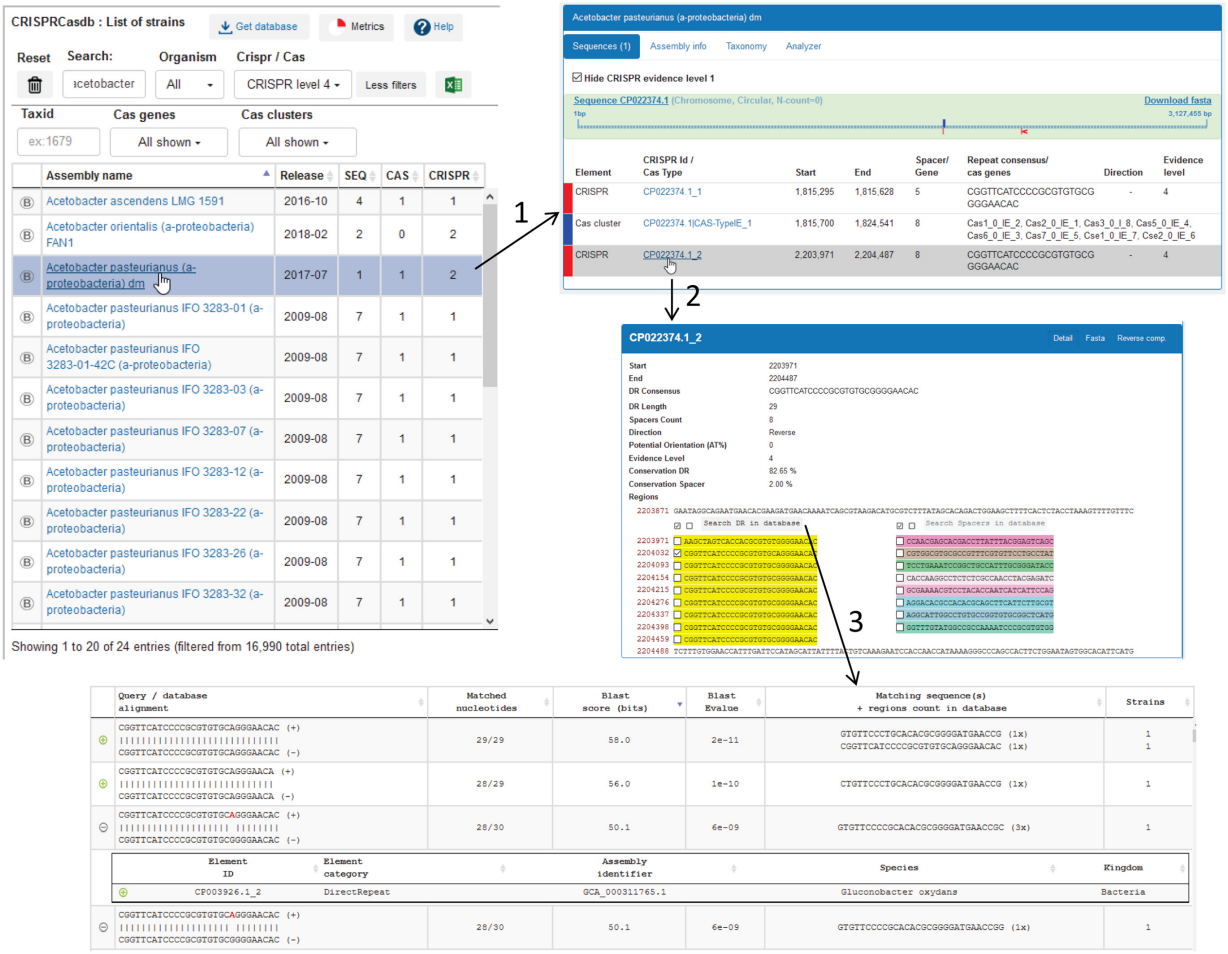
Screenshots of the browse page and output in CRISPRCasdb. (1) Selecting a strain leads to a schematic representation of the genome with the position of CRISPR arrays and *cas* clusters present in the genome(s) and a table with their position, sequence of the consensus repeats and name of *cas* genes. (2) The CRISPR array is depicted with repeats coloured in yellow and spacers with different colours. (3) Selected sequences (repeat or spacer) can be blasted against lists present in the database.

The strains can be displayed as an alphabetical list or by taxonomy, allowing observation of CRISPR–Cas systems in phylogenetically related species, and search can be performed by strain name or GenBank/RefSeq accession number. ‘Metrics’ provides the date of the last update and global statistics on the number of Cas clusters and level 4 CRISPR arrays. The list of strains can be filtered on the basis of CRISPR or *cas* number or on the presence of CRISPRs with evidence levels 1 to 4, species Taxid, Cas name and CRISPR–Cas type. Figure 2 details some of the pages which can be viewed when selecting a strain within the alphabetical order view. After clicking on the strain name (step 1), a table on the right appears, showing for each genome present in the strain (chromosome or plasmid) the CRISPRs and *cas* genes clusters that have been found. Authorizing small CRISPR-like structures in the database sometimes leads to an important amount of evidence level 1 arrays. For convenience they can be hidden to facilitate observation of the most interesting structures. The CRISPR–Cas type and subtype is indicated together with the *cas* genes cluster position on the genome, the number of spacers or *cas* genes and the consensus repeat sequence. An indication of the repeat orientation (when known) and of the CRISPR evidence level is shown. On top of the page, a schematic representation of the genome displays the positions of the CRISPR arrays and *cas* genes. An arrow appears when an element is selected. Querying a CRISPR locus (step 2) leads to a page containing a graphical representation together with sequence retrieval tools. The repeat sequence is shown in yellow, the spacers are shown in different colours together with their position in the genome, and 100 bp of flanking sequence are provided. The CRISPR sequence and the list of spacers in Fasta format can be uploaded allowing analyses with other bioinformatic resources. Furthermore it is possible to perform a BLAST search of one or several repeats or spacers against the complete list of level 4 repeats and of spacers present in the database (step 3 and following paragraph).

### The repeats and spacers lists

Repeat sequences of all the evidence level 4 CRISPRs from the database are listed and can be downloaded as a fasta file. Presently they amount to 19 321 sequences. For each repeat the id of the CRISPR in which the sequence was observed is indicated. Similarly, a list of 211 397 spacers encountered in level 4 CRISPRs is available. A dedicated page called ‘search DR/spacers’ allows running a BLAST (blastn) using selected spacers or repeats against these lists with a default cutoff E-value of 0.1 and a matching length of at least 70% the queried sequence size. A maximum of 200 results per sequence queried is displayed and the mismatches are shown in red (Figure 2, bottom panel). Clicking anywhere on a line showing the alignment leads to the name of the target genome in which it is present and display of the corresponding CRISPR. A BLAST search can also be sent from CRISPRs stored in the database or following a CRISPRCasFinder search.

### Distribution of CRISPR–Cas systems

**Figure 3.**
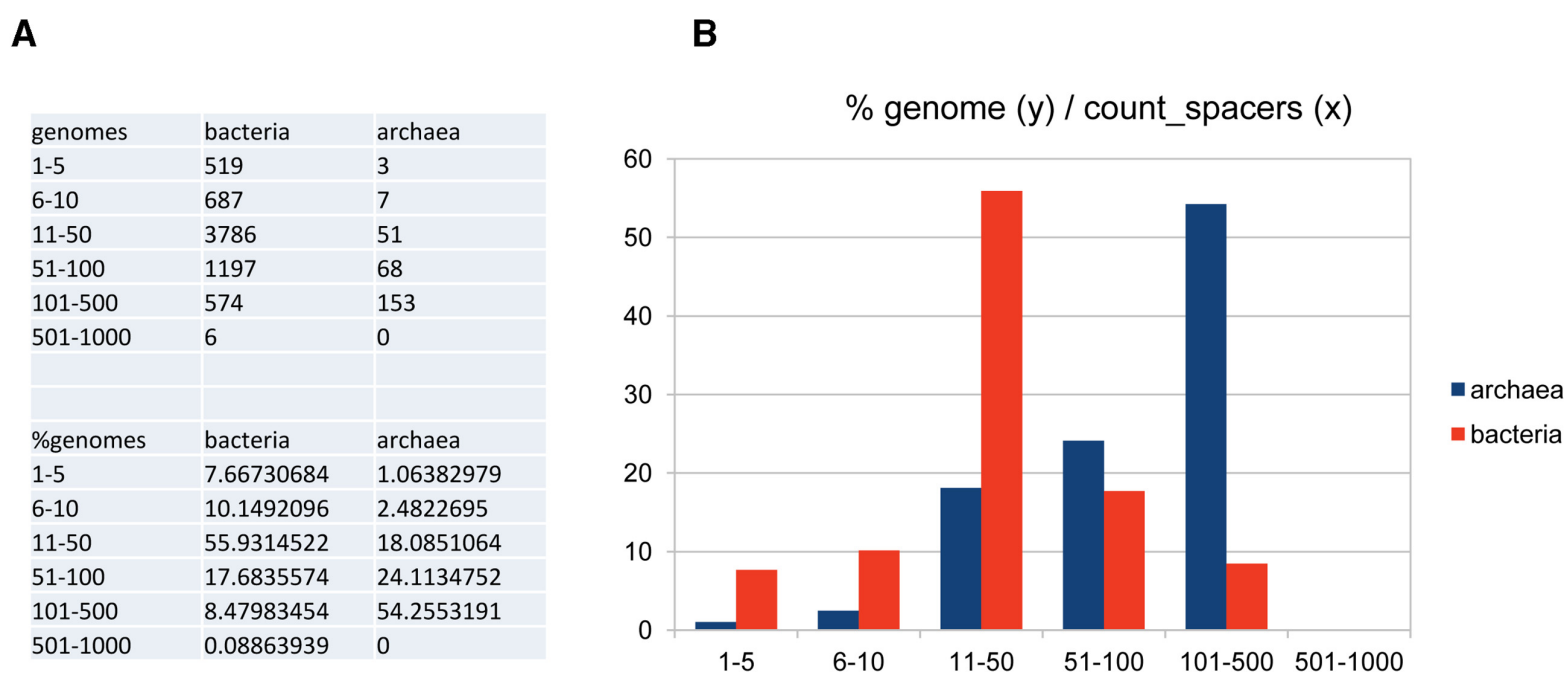
Evaluation of total number of spacers per strain. (**A**) Total number of spacers present in 6769 bacterial genomes and 282 archaeal genomes. (**B**) Genomes are distributed in percentage in function of the number of spacers.

CRISPRCasdb has been constructed using public complete genome sequences (unpublished sequences can be analysed *via* the CRISPRCasFinder page). A large proportion of the structures qualifying as CRISPRs using the defined parameters possess four or less than four spacers and the majority of these are classified as level 1 and level 2. However, if they show a repeat present in a level 4 CRISPR, they will be upgraded and shown as level 4 in CRISPRCasdb. Among Bacteria and Archaea, respectively 36% and 75.3% have at least one *cas* genes cluster and one level 4 CRISPR (Table 1). These percentages are somewhat biased by the differential representation of sequenced strains inside species but, due to the wide genetic diversity inside some species, it is impossible to select a single representative strain. Most importantly the percentages are based on the bacteria and archaea which genome has been fully sequenced and therefore cannot be representative of the full microbial diversity. A total of 622 genomes (about 4%) belonging to 240 species in 125 genera have no *cas* genes cluster but at least one level 4 CRISPR. In the majority of cases some or all the *cas* genes necessary to make a functional cluster are indeed absent and therefore the cluster is not identified by the Subtyping model of Cas-Finder. In some strains putative Cas may not be detected because they do not present sufficient similarities with proteins used to derive the HMM models. Finally, a few percent of strains have *cas* genes clusters with no detectable CRISPR (Table 1). In Bacteria 546/15938 (3.4%) plasmids possess a level 4 CRISPR whereas in Archaea the ratio of plasmids carrying a CRISPR is 30/179 (16.7%). The longest CRISPR with 587 spacers is found in the Bacteria *Haliangium ochraceum* strain DSM 14365 (GCA_000024805). Interestingly, Archaea have statistically a larger number of spacers in total, distributed into several CRISPR arrays as shown on Figure 3 and Supplementary Figure S4. More than 50% of archaeal genomes have a total of 100 or more spacers. Given the generally small size of archaeal genomes this reflects the importance of CRISPR–Cas systems in Archaea. In Bacteria, the majority of genomes have a total of 50 spacers or less. Supplementary Figure S5 shows that no correlation can be made between the size of bacterial or archaeal genome and the total number of spacers.

**Table 1.** Distribution of level 4 CRISPR arrays and of *cas* clusters (CAS) in Bacteria and Archaea

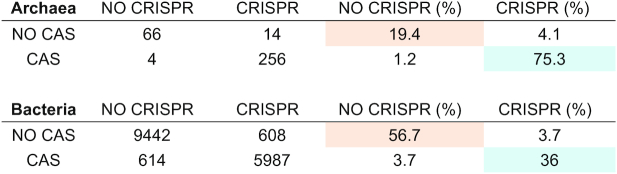

**Figure 4.**
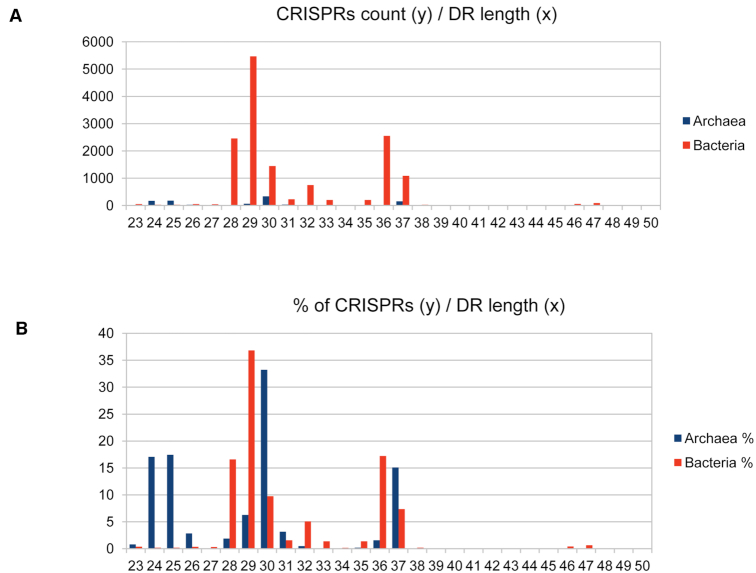
Repeat size distribution. (**A**) *x* is the repeats size in bp and *y* is the number of CRISPR arrays. (**B**) *x* is the repeats size in bp and *y* is the percentage of CRISPR arrays.

**Figure 5.**
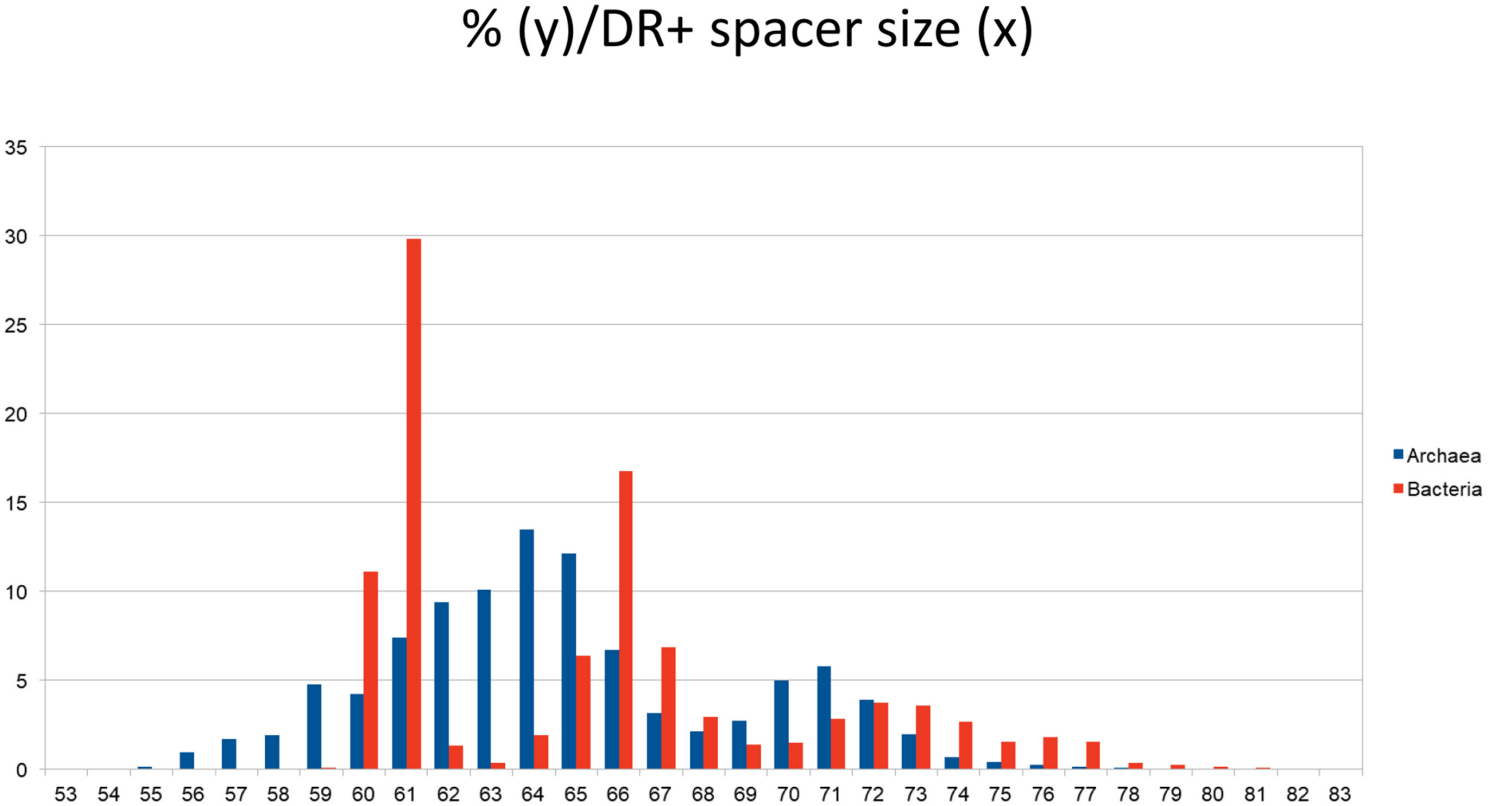
Relative size of spacers and repeats. *x* is the total size of ‘repeat + spacer’ and *y* is the percentage of size occurrence.

Wide differences are observed among the CRISPRs, in the repeat sequence, its size and the size of the spacers. The diagrams on Figure 4 show the total number (A) or the percentage (B) of CRISPR arrays with repeat size from 23 to 50 bp. A similar pattern is seen when the distribution of DR size is shown according to species (Supplementary Figure S6). As previously observed on a smaller amount of genomes in both Archaea and Bacteria, three major size classes are observed with repeat of small (24–25 bp), medium (28–30 bp) and large size (36–37 bp). Archaea tend to possess CRISPRs with small repeats whereas those with the largest repeat size, >40 bp are found only in Bacteria and are mostly associated with Cas type II systems. By calculating the respective sizes of repeats and spacers we find that the size range of repeat plus spacer is 55–81 bp with a peak in the range 60–67 bp (Figure 5).

**Figure 6.**
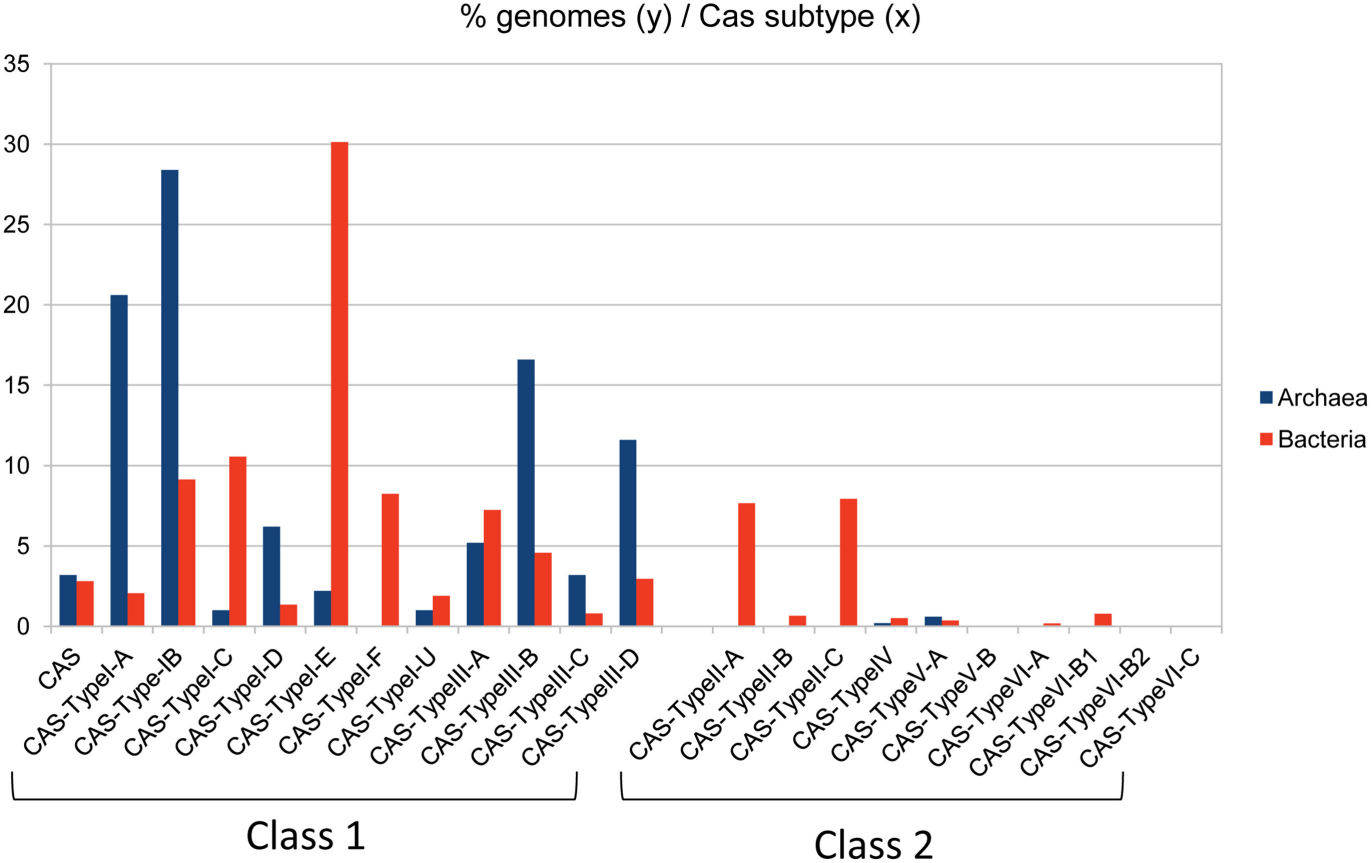
Number of Cas types and subtypes. The different CRISPR–Cas subtypes are shown on the x axis and the percentage of genomes are shown on the y axis.

Figure 6 shows the distribution of systems’ type and subtype while Supplementary Table S1 shows details on types combinations inside strains. With the exception of three strains, only type I and type III systems are found in Archaea, type I-B being the most abundant. A class 2 type V-A system was observed in *Methanoplasma termitum* strain MpT1 and in two strains of *Methanomethylophilus alvus*, Mx-05 and Mx1201, with CRISPRs possessing a closely associated repeat. Type V-A systems possess a long Cas protein (Cas12/Cpf1) which performs multiple functions similarly to type II Cas9 (37). The Cas12 of these three strains showed 39% identity, as low as with the reference bacterial Cpf1 proteins from *Francisella novicida*. In Bacteria, type I systems (in particular type I-E) are the most abundant and type IV, V and VI are rare.

In Archaea, type I and type III are often associated and co-localized as shown on Supplementary Figure S7 for *Metallosphaera sedula* ARS120. In total, 123 out of 142 type III-B systems are associated with a type I-A, type I-B or type I-C system. Such associations are also observed in bacteria not only with type I systems but also with other Cas types (Supplementary Table S1). It was proposed that type I and type III cooperate to counteract viral escape in *Marinomonas mediterranea* (38).

The analysis of large numbers of strains from a single species confirms that some species or genus seem to be completely or mostly devoid of CRISPR–Cas systems. For example in *Staphylococcus aureus* only five strains out of 449 possess a type III-A system. In the genus *Bordetella* with a total of 637 strains analysed in the database, only three possess a CRISPR–Cas system. Intracellular organisms such as *Chlamydia*, *Rickettsia* and *Brucella* have no CRISPR–Cas systems. By contrast Thermophilic bacteria and cyanobacteria often possess several CRISPR–Cas systems (mostly combinations of type I and type III systems) with multiple CRISPRs, some of them being very long. This is the case of *Sphaerospermopsis kisseleviana* NIES-73 with nine *cas* clusters, or of all the members of the *Caldicellulosiruptor* genus with up to six different *cas* clusters.

### Comparison with other databases

We could identify four additional web-based databases for CRISPR arrays and *cas* genes (Table 2). CRISPI (https://crispi.genouest.org/) is mostly dedicated to the identification of CRISPR arrays whereas CRISPRBank, CRISPRone and CRISPRminer use different programs to define CRISPR and *cas* clusters, which results in a number of differences with CRISPRCasdb. We previously discussed the performances of the CRISPR and *cas* genes identification tools in the different programs and concluded that none achieved a perfect identification of both (28). Accordingly when comparing the databases we could observe a number of discrepancies in characterization of CRISPR arrays and definition of *cas* gene clusters, although overall these databases can be complementary in providing a thorough investigation of CRISPR–Cas systems. Improvements of CRISPRFinder are regularly made to obtain a more accurate definition, the last version including the possibility to validate small CRISPRs when their repeat belongs to a level 4 CRISPR. Such small CRISPRs are not taken into account in the other databases.

**Table 2.** Comparison between CRISPRCasdb and previously established databases for CRISPR arrays and Cas

Name	Date issue	Last update	Nbr Bacteria	Nbr Archaea	CRISPR detection	Cas detection	System type
CRISPRdb	2007	2017/05/09	6782*	232*	CRISPRFinder	no	no
CRISPI	2009	2017/03/21	2644	168	Pygram	BLAST	no
CRISPRBank	2016		2571*	162*	CRISPRDetect	GenBank annotation	yes
CRISPRone	2017	2018/08/01	32 288		MetaCRT	HMM search	yes
CRISPRminer	2018	2017	43 140	167	PILER-CR	HMM search	yes
CRISPRCasdb	2018	2019/06/17	16 650*	340*	CRISPRFinder	HMM search	yes

*Complete genomes only.

The web sites also differ in the interface and tools offered. CRISPRBank (http://bioanalysis.otago.ac.nz/CRISPRBank/) possesses CRISPR arrays and *cas* genes from 2733 strains and can be accessed by querying repeats and spacers. CRISPRminer (http://www.microbiome-bigdata.com/CRISPRminer) provides a database of CRISPR and *cas* genes from complete and draft prokaryote genomes, and additional information on self-targeting, anti-CRISPR genes and the nature of protospacers. Genomes can be browsed by providing the name of a strain or RefSeq ID or using taxonomic classification. CRISPRone provides a long list of ‘mock CRISPRs’, most of which are not detected by CRISPRFinder or by Tandem Repeat Finder (TRF) (39), which raises questions on their relevance. CRISPRone (http://omics.informatics.indiana.edu/CRISPRone/), like CRISPRminer displays CRISPR arrays and *cas* genes in a graphical fashion and provides different files with details on repeats and spacers.

Presentation in CRISPRCasdb of analysed genomes in the form of an alphabetical list of strains or taxonomic classification is particularly useful to browse the database and discover potentially interesting CRISPR–Cas systems. This interface is not proposed in the other databases available on the web as one must indicate the name or accession number of the organism to query. In CRISPRCasdb the possibility to apply filters such as on the CRISPR arrays evidence levels or presence/absence of Cas allows to select the most relevant information for a given purpose.

## DISCUSSION AND FUTURE PROSPECTS

CRISPRCasdb by allowing the identification of both CRISPRs and *cas* genes is a major improvement over CRISPRdb and will replace it once users are fully aware of the new database. We first chose to analyse only complete genomes because we were concerned by the correct identification of CRISPR–Cas systems, which requires that the full *cas* gene cluster be present on a single sequence. In the future, data extracted from draft or large contigs will be included with an indication of their nature. Following the first analyses by Banfield's group (40) detection of CRISPR arrays in metagenomics data has been performed by different teams and specific tools were developed such as Crass (41), MinCED (https://github.com/ctSkennerton/minced) and MetaCRAST (42). It will be an interesting challenge to propose such tools on CRISPR-Cas++ owing to the considerable size of data to be processed and required calculation time.

Some functions offered together with CRISPRdb will be implemented, such as a tool to compare alleles of a given CRISPR array among strains, and classify the spacers. Several programs are available such as CRISPRtionary (43), CRISPR Visualizer (44) and CRISPRStudio (45) for comparative visualization of CRISPR content. The new CRISPR–Cas++ website has high performances and flexibility which will allow further evolutions. Its architecture was designed to allow additional developments and in particular the possibility to evolve toward a real webserver by proposing an Application Programming Interface (API). This will give the possibility to interact programmatically with the database and the different applications.

We performed some analyses on the database content to illustrate its potential use but more need to be done to understand the distribution of CRISPR–Cas systems in prokaryotic genomes. The resource was developed to allow such investigations by the scientific community. Our results confirmed observations made with a smaller number of strains and highlighted some characteristics previously described such as the absence of CRISPR–Cas systems in some species and genera (33). Among strains possessing level 4 CRISPRs and no complete *cas* gene cluster, undetected *cas* genes might be present. We observed putative Cas9-like proteins that are highly divergent from the known reference proteins and which need to be further investigated. To better identify such proteins, the HMM models must be continuously adapted to the discovery of new Cas9-like sequences (based on the presence of HNH-4 and RuvC-III domains). It will be interesting to analyse the spacer diversity and distribution in various groups as well as across CRISPR-Cas types as it may reflect activity and point to groups that are of particular relevance for future studies.

There are still unexplained phenomena including the existence in some strains of multiple CRISPR arrays with the same or similar repeat and with different spacers. Crawley *et al.* proposed to call ‘Split arrays’ CRISPRs that are not localized near the *cas* gene cluster but possess the same repeat as the main array (33). However there is no evidence on the mechanism of creation of such arrays that do not share any spacers but possess a common leader. The large spectrum of CRISPR–Cas systems confirms the complex relationship between microorganisms and their environment and the relative importance of the CRISPR–Cas immune system as a defence mechanism.

## DATA AVAILABILITY

The resource described here is accessible with no restrictions, except for the demand to quote the site.

## Supplementary Material

gkz915_Supplemental_Files

## FUNDINGS

Institut Français de Bioinformatique (IFB) [ANR-11-INSB-0013]. Funding for open access charge: CNRS.
