# Supplementary material for: CRISPRCasdb a successor of CRISPRdb containing CRISPR arrays and cas genes from complete genome sequences, and tools to download and query lists of repeats and spacers
Source: Nucleic Acids Res. 2019 Oct 18;48(D1):D535–44. doi: 10.1093/nar/gkz915 (PMC7145573; doi:10.1093/nar/gkz915)
Supplement: gkz915_Supplemental_Files [file gkz915_supplemental_files.zip › Supplementary file.pdf]

I-Database and software design and implementation

Figure S1: WORKFLOW

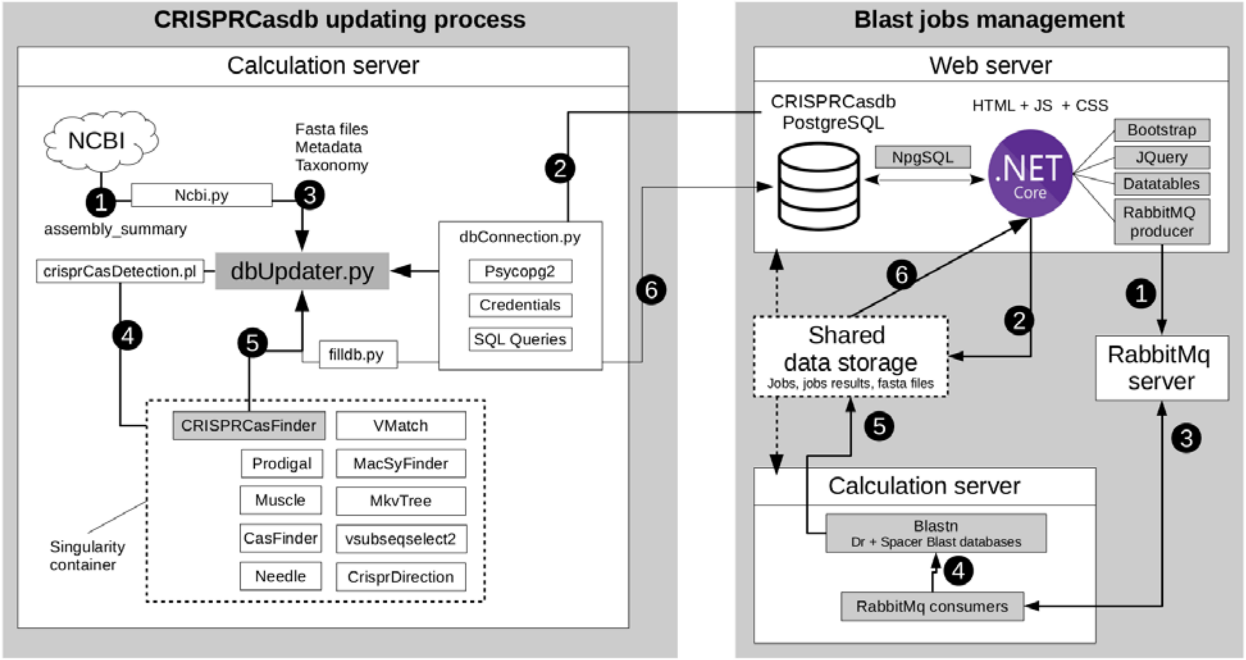

Legend to WORKFLOW

CRISPRCasdb updating process: the main calculation server of CRISPRCAS++ project is used to update the database. The development of a weekly automatic updating process is planned in the next version of CRISPRCasdb updater. (1) The updater main script downloads the assembly_summary file for bacteria and archaea. We solely consider assemblies with completed genomes (column assembly_level = "Complete genome"). (2) The updater checks for new assemblies comparing database release dates and assemblies names, and then (3) downloads new assemblies with strain metadata and taxonomy. Outdated assemblies are deleted from the database. (4) Each downloaded assembly is then analyzed using the latest CRISPRCasFinder version and stored in a Singularity container with all its dependencies. (5) CRISPRCasFinder

produces a JSON result file, containing CRISPR array and *cas* cluster detection results. (6) The updater script inserts these results in the database, with strain metadata and taxonomy.

BLAST jobs management: the CRISPRCas++ web server hosts the web application frontend, and the PostgreSQL database. The main calculation server of CRISPRCAS++ hosts the blastn program, and the RabbitMQ consumer's service. (1) When a user submits a new Blast job, the web application sends the job ID to the RabbitMq server for job queuing. (2) Simultaneously, an archive containing the sequence(s) to blast and the job's parameters is dropped on a storage space shared between the web server and the calculation server. (3) A RabbitMQ consumer receives the job ID and opens the corresponding job archive on the storage space. The job is then deleted from RabbitMQ queue. Multiple RabbitMQ consumers can be launched for multiprocessing. (4) The RabbitMq consumer runs Blastn on all submitted sequences, against lists of direct repeats and spacers. (5) The Blast results are stored into a JSON file, (6) read by the web application that will display each Blast result for each analyzed sequence.

II- Composition of the database

Figure S2: UML class diagram

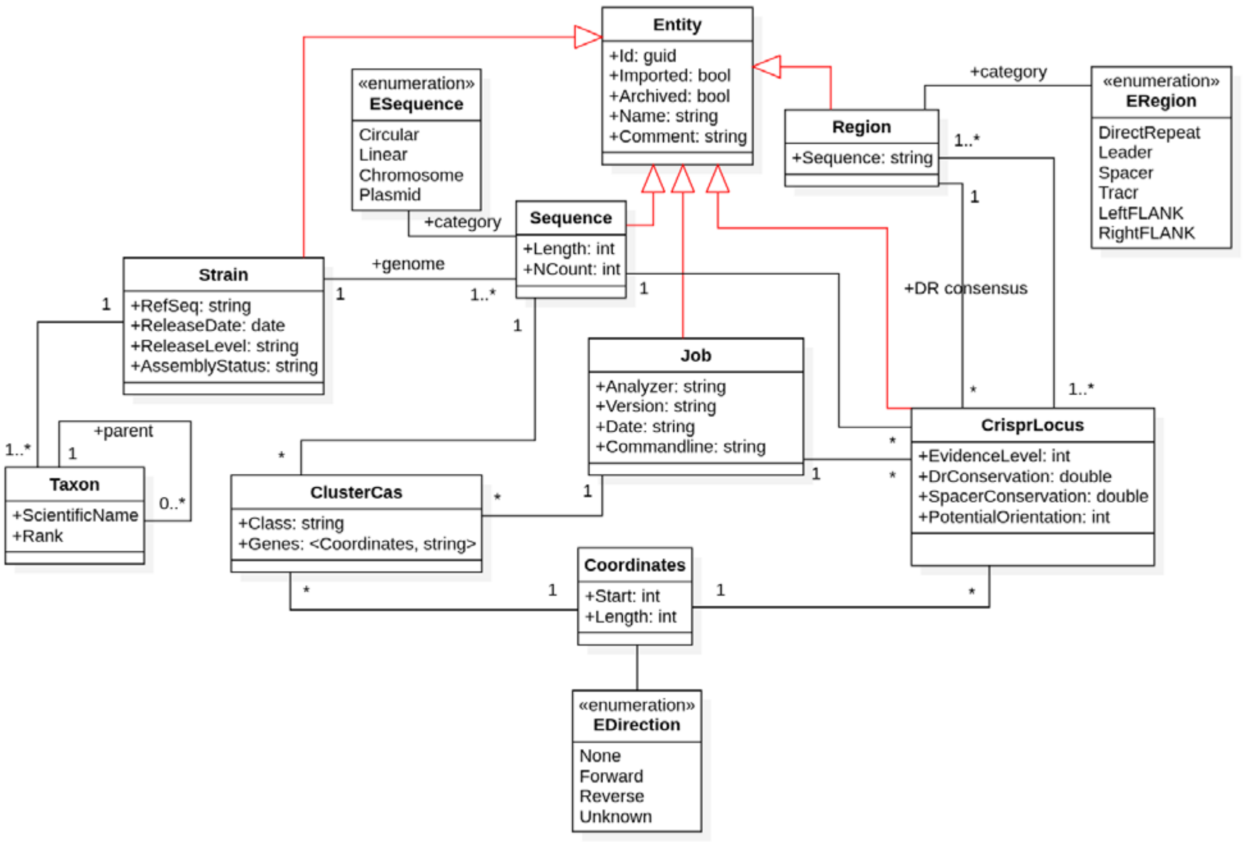

Legend to UML class diagram

Entity: the parent class of most other classes stores the name of the child classes, comments etc.. and methods common to other classes,

Strain: assemblies metadata (RefSeq and GenBank accessions (ex: GCA_000258245.1), release level ("Major, Minor"), assembly status ("latest"),

Sequence: metadata for each sequence of a strain; length of each sequence, count of N in sequence. The complete nucleotide sequences are not stored in the database, but in fasta files (for *cas* gene sequence viewing),

CrisprLocus (+**Coordinates**): position of each detected CRISPR locus, evidence level (1 to 4), direct repeat consensus sequence (in **Region** class), direct repeats conservation level, spacers conservation level, orientation of CRISPR,

Region: ordered list of sequences; Direct repeats (inc. consensus DR), leader, spacers, Tracr, left flanks, right flanks,

ClusterCas (+**Coordinates**): position of each detected *cas* genes cluster, Type and Subtype of Cas cluster (ex : TypeIE), and names of HMM profiles (cas 1, 2, 3…),

Taxon: Complete taxonomy for each strain; taxonomic ranks (genus, species…), and associated name (Escherichia, Escherichia coli).

Figure S3: Tables' interaction

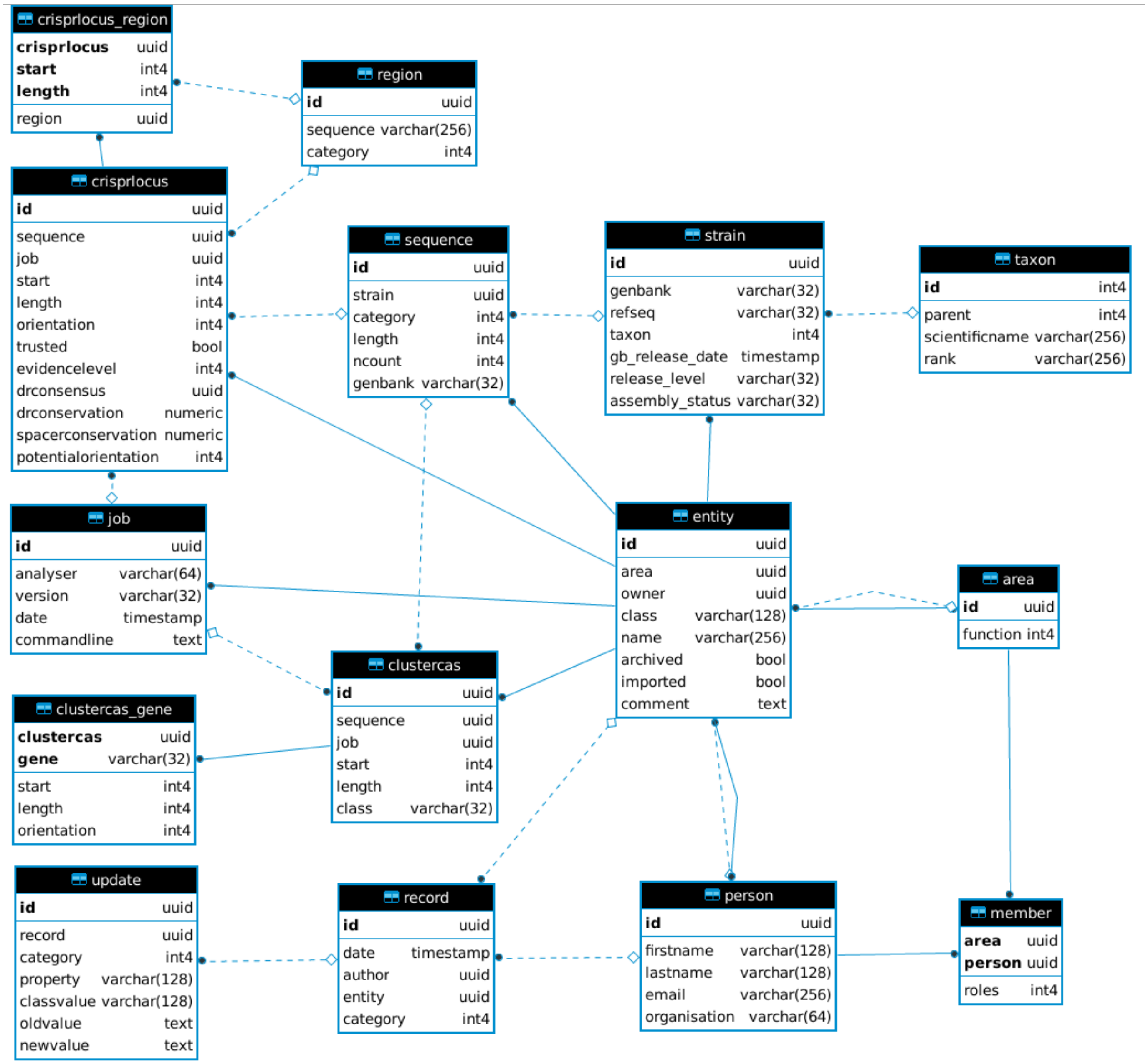

III-Statistics

Figure S4: Statistical evaluation of the total number of spacers in Bacteria and Archaea complete genomes. The T-test P-value is <2.2e-16

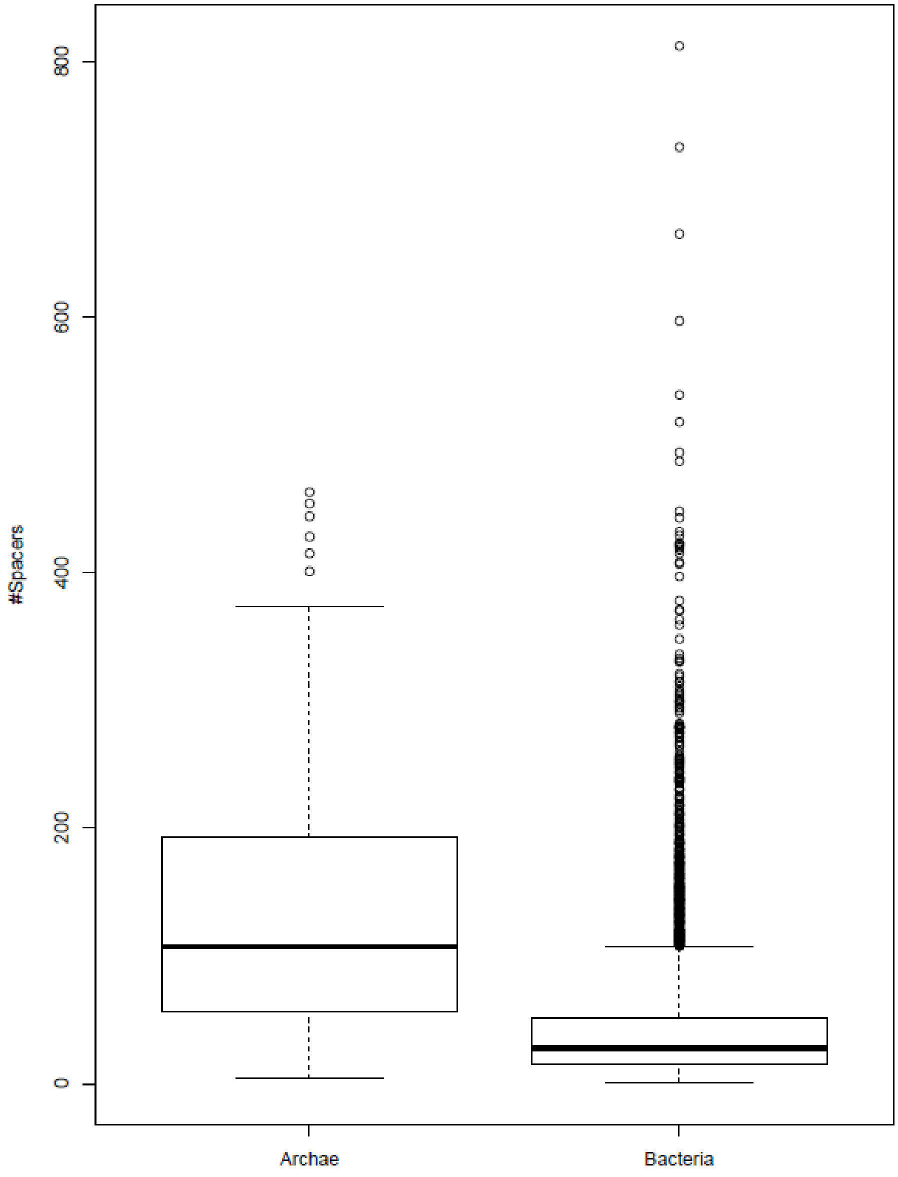

Figure S5: Distribution of spacers according to the genome size

Archaea

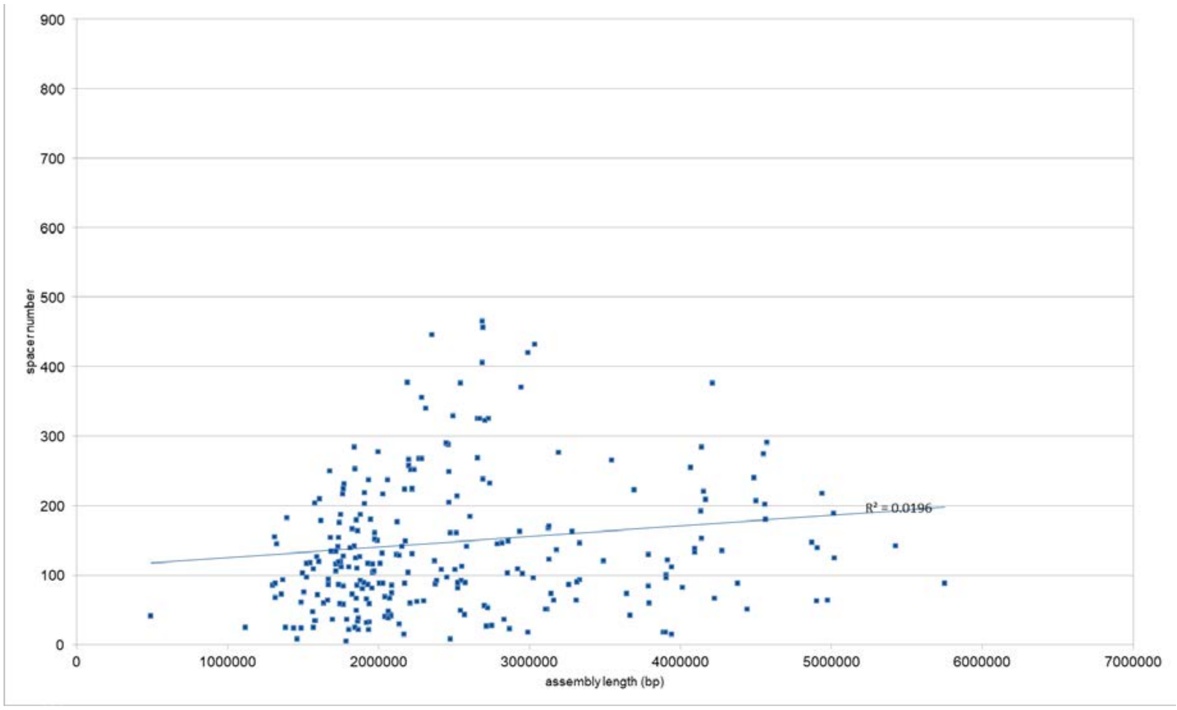

Bacteria

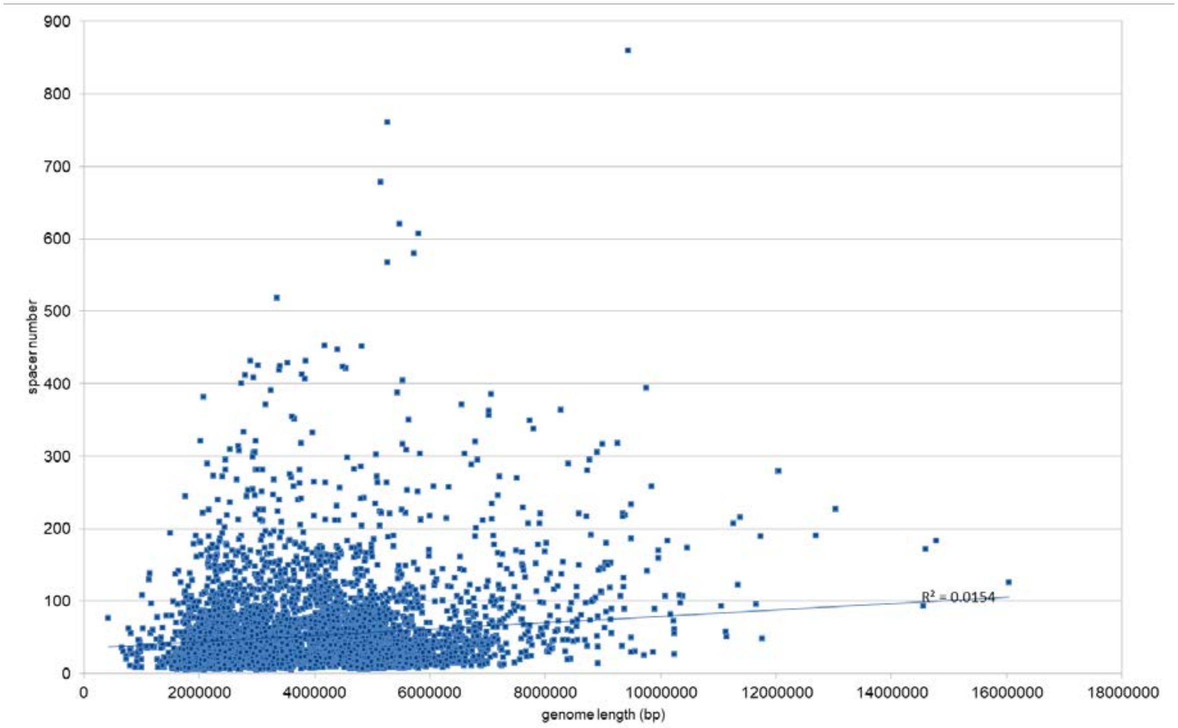

Figure S6: Repeat size distribution according to species

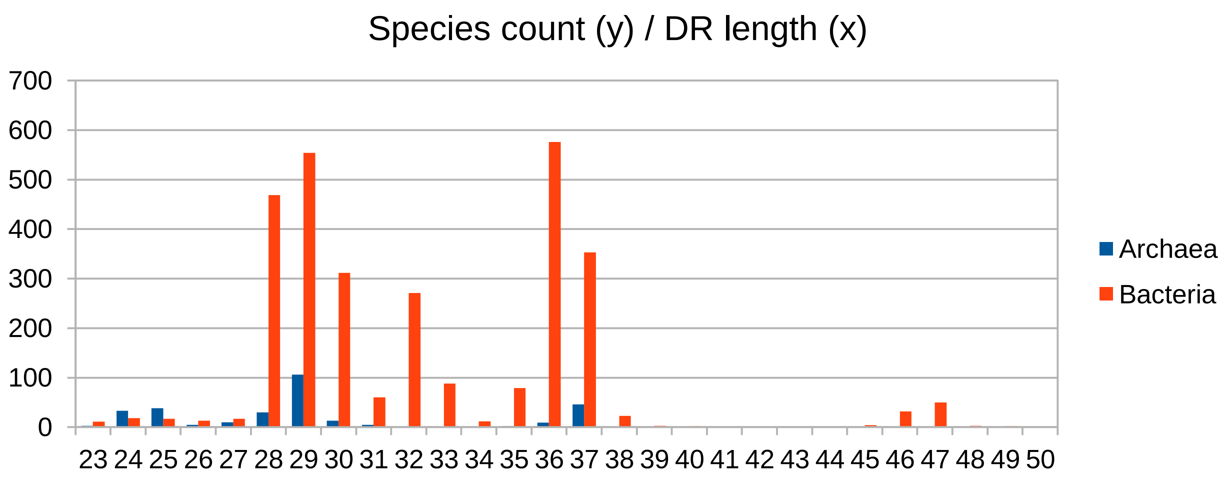

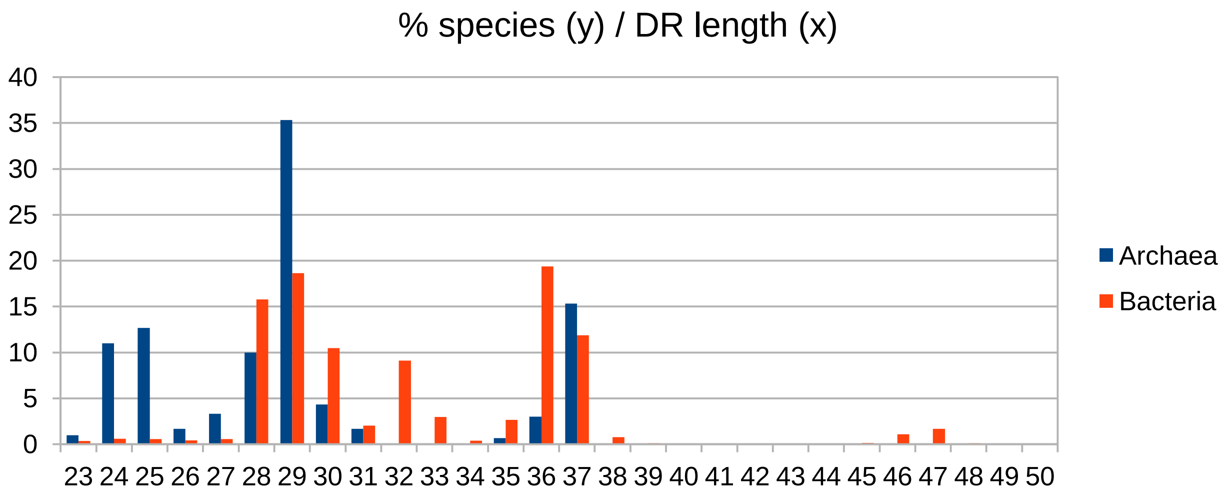

IV-Localization of CRISPR-Cas systems

Figure S7: Co-localization of Type I and Type III systems

CRISPRs and *cas* genes are intermixed into a large locus in *Metallosphaera sedula*

Metallosphaera sedula (crenarchaeotes) ARS120-1

Sequences (1) Assembly info Taxonomy Analyzer

☑ **Hide CRISPR evidence level 1**

Sequence CP012174.1 (Chromosome, Circular, N-count=0) Download fasta

1bp 2,191,531 bp

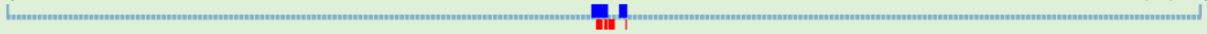

Element	CRISPR Id / Cas Type	Start	End	Spacer/ Gene	Repeat consensus/ cas genes	Direction	Evidence level
Cas cluster	CP012174.1|CAS-TypeIA_1	1,073,313	1,102,152	12	Cas1_0_I-II-III_9, Cas2_0_I-II-III-V_10, Cas3_0_I_4, Cas3_1_I_3, Cas4_0_IA_8, Cas4_0_I-II_11, Cas5_0_IA_5, Cas6_0_I-III_1, Cas6_0_I-III_12, Cas7_0_IA_6, Csa5_0_IA_7, CsaX_0_IA_2		
CRISPR	CP012174.1_4	1,082,256	1,091,910	150	CTTTCAACTCTATAGGAGATTAAC	+	4
CRISPR	CP012174.1_5	1,097,327	1,100,889	55	CTTAATCTCCTATAGAGTTGAAAG	-	4
CRISPR	CP012174.1_6	1,104,341	1,114,686	161	GTTAATCTTCTATAGAGTTGAAAG	-	4
Cas cluster	CP012174.1|CAS-TypeIIID_2	1,124,105	1,137,617	8	Cas10_0_III_4, Cas3_1_I_8, Cas6_0_I-III_7, Csm3_0_IIID_3, Csm3_1_IIIAD_1, Csm3_1_IIIAD_2, Csm3_1_IIIAD_6, Csm3_1_IIID_5		
CRISPR	CP012174.1_7	1,136,108	1,136,623	7	GTTTCAAACCCTCGAAGGATCTCTA CAAAC	ND	4
